# Overview of the pathological results and treatment characteristics in the first 1000 patients randomized in the SERC trial: axillary dissection versus no axillary dissection in patients with involved sentinel node

**DOI:** 10.1186/s12885-018-5053-7

**Published:** 2018-11-21

**Authors:** Gilles Houvenaeghel, Monique Cohen, Pédro Raro, Jérémy De Troyer, Christine Tunon de Lara, Pierre Gimbergues, Tristan Gauthier, Christelle Faure-Virelizier, Véronique Vaini-Cowen, Stéphane Lantheaume, Claudia Regis, Emile Darai, Vivien Ceccato, Gauthier D’Halluin, Francesco Del Piano, Richard Villet, Eva Jouve, Bassoodéo Beedassy, Pierrick Theret, Philippe Gabelle, Cécile Zinzindohoue, Pierre Opinel, Catherine Marsollier-Ferrer, Caroline Dhainaut-Speyer, Pierre-Emmanuel Colombo, Eric Lambaudie, Agnès Tallet, Jean-Marie Boher, Others investigators (SERC trial group), Nicolas Sterkers, Jean-Claude Darmon, Julia Pernaut, Xavier Martin, Aubert Agostini, Max Buttarelli, Emile Mereb, Jean-François Le Digabel, Sophie Girard, Sandra Houlard, Ludivine Genre, Jean-Marie Brandone, Théophile Hoyek, Issam Baalbaky, Ludivine Loussert, Brice Gurriet, Renaud Haberstich, Christiane Pourny, Olivier Audrin, Gilles Carrasset, Frédéric Caquant, Jean-Philibert Combier, Frédéric Dedecker

**Affiliations:** 1 Institut Paoli Calmettes & CRCM & Aix Marseille Univ, 232 Bd de Sainte Marguerite, 13009 Marseille, France; 2 Department of surgery, Institut Paoli Calmettes & CRCM & Aix Marseille Univ, 232 Bd Ste Marguerite, Marseille, France; 3 Department of radiotherapy, Institut Paoli Calmettes & CRCM & Aix Marseille Univ, 232 Bd Ste Marguerite, Marseille, France; 4 Department of biostatistics, Institut Paoli Calmettes & CRCM & Aix Marseille Univ, 232 Bd Ste Marguerite, Marseille, France; 5 Institut de Cancérologie de l’Ouest - Site Paul Papin, 15 rue André Boquel, 10059 49055 Angers Cedex 02, CS France; 6 Polyclinique Urbain V, Chemin du Pont des Deux Eaux, 84000 Avignon, France; 7 Institut Bergonie, 229 cours de l’Argonne, 33076 Bordeaux Cedex, France; 8 Centre Jean Perrin, 58 rue Montalembert BP 392, 63011 Clermont Ferrand Cedex, France; 9 HME CHU Dupuytren, 2 avenue Martin Luther King, 87000 Limoges, France; 10 Centre Léon Bérard, 28 rue Laennec, 69373 Lyon Cedex 8, France; 11 Clinique du Parc Rambot 2, Avenue du Dr Aurientis, 13100 Aix en Provence, France; 12 Clinique Pasteur, 294 boulevard Charles de Gaulle, 07500 Guilherand Granges, France; 13 Centre Oscar Lambret, 3 rue F. Combemal, 59000 Lille, France; 14 Hôpital Tenon, 4 rue de la Chine, 75020 Paris, France; 15 Institut Jean Godinot, 1 rue du Général Koenig, 51056 Reims, France; 16 Centre Clinical, 2 chemin Frégenueil CS 42510 Soyaux, 16025 Angoulème, France; 17 Hôpitaux Du Léman, 3 avenue de la Dame, 74200 Thonon, France; 18 Groupe Hospitalier Des Diaconesses Croix Saint Simon, Site Reuilly, 18 rue Sergent Bauchat, 75012 Paris, France; 19 Institut Universitaire du Cancer Toulouse, Oncopole, 1 avenue Irène Joliot-Curie, 31059 Toulouse, France; 20 Hôpital Sainte Musse (CHITS), Service de chirurgie viscérale, Rue Henri Sainte-Claire Deville, 83056 Toulon, France; 21 CH Saint Quentin, 1 avenue Michel de l’Hospital, B.P. 608, 02321 Saint Quentin Cedex, France; 22 GHM de Grenoble, La Clinique des Eaux Claires, 8 rue du Dr Calmette, 38028 Grenoble Cedex 1, France; 23 Clinique Clementville, 25 rue de Clémentville, 34070 Montpellier, France; 24 CHR du Pays d’Aix, Avenue des Tamaris, 13616 Aix en Provence Cedex 1, France; 25 CHRU Nimes, Place du Pr Robert Debré, 30029 Nimes Cedex 9, France; 26 GCS Recherche et Innovation Sante Sarcelles, 6 avenue Charles Peguy, 95200 Sarcelles, France; 27 ICM – Institut Régional du Cancer Montpellier, 208 avenue des Apothicaires – Parc Euromédecine, 34298 Montpellier Cedex 5, France

**Keywords:** Breast cancer, Sentinel lymph node biopsy, Axillary lymph node dissection, Randomized trial

## Abstract

**Background:**

Three randomized trials have concluded at non inferiority of omission of complementary axillary lymph node dissection (cALND) for patients with involved sentinel node (SN). However, we can outline strong limitations of these trials to validate this attitude with a high scientific level. We designed the SERC randomized trial (ClinicalTrials.gov, number NCT01717131) to compare outcomes in patients with SN involvement treated with ALND or no further axillary treatment. The aim of this study was to analyze results of the first 1000 patients included.

**Methods:**

SERC trial is a multicenter non-inferiority phase 3 trial. Multivariate logistic regression analysis was used to identify independent factors associated with adjuvant chemotherapy administration and non-sentinel node (NSN) involvement.

**Results:**

Of the 963 patients included in the analysis set, 478 were randomized to receive cALND and 485 SLNB alone. All patient demographics and tumor characteristics were balanced between the two arms. SN ITC was present in 6.3% patients (57/903), micro metastases in 33.0% (298), macro metastases in 60.7% (548) and 289 (34.2%) were non eligible to Z0011 trial criteria.

Whole breast or chest wall irradiation was delivered in 95.9% (896/934) of patients, adjuvant chemotherapy in 69.5% (644/926), endocrine therapy in 89.6% (673/751) and the proportions were similar in the two arms. The overall rate of positive NSN was 19% (84/442) for patients with cALND. Crude rates of positive NSN according to SN status were 4.5% for ITC (1/22), 9.5% for micro metastases (13/137), 23.9% for macro metastases (61/255) and were respectively 29.36% (64/218), 9.33% (7/75) and 7.94% (10/126) when chemotherapy was administered after cALND, before cALND and for patients without chemotherapy.

**Conclusion:**

The main objective of SERC trial is to demonstrate non inferiority of cALND omission. A strong interaction between timing of cALND and chemotherapy with positive NSN rate was observed.

**Trial registration:**

This study is registered with ClinicalTrials.gov, number NCT01717131 October 19, 2012.

**Electronic supplementary material:**

The online version of this article (10.1186/s12885-018-5053-7) contains supplementary material, which is available to authorized users.

## Key message

SERC trial compared outcomes in patients with SN involvement treated with ALND or no further axillary treatment. We analyze results of the first 1000 patients included. Crude rates of positive NSN according to SN status were 4.5, 9.5, 23.9% for ITC, micro and macro metastases and were respectively 29.3, 9.3 and 7.9% when chemotherapy was administered after or before cALND and without chemotherapy.

## Background

The firsts randomized trials confirmed that sentinel lymph node biopsy (SLNB) accurately staged the axilla if sentinel nodes (SN) were not involved [1–3]. If SN was involved, standard practice was complementary axillary lymph node dissection (cALND) [4]. Axillary recurrences are rare, even with omission of ALND [5, 6]. The main use of axillary surgery is a disease staging procedure and ALND may have a favorable effect on survival [2]. However, the side-effects of ALND including lymphedema, pain and reduced arm movement are higher in comparison with SLNB alone [1, 7–9].

SLNB provide information to guide adjuvant treatments complementary to tumor characteristics and particularly molecular subtypes. However, the entire SN serial section examination eventually with cytokeratin immunostaining resulted in the frequent identification of small SN involvement, isolated tumor cells (ITC) or micro-metastases.

Three randomized trials have concluded at non inferiority of cALND omission for patients with involved SN [9–11]. These results conduct to propose to avoid cALND [12, 13] for patients with all criteria reported in these trials. However, we can outline strong limitations of these trials to validate this attitude with a high scientific level.

We designed the SERC trial to compare outcomes in patients with SN involvement treated with ALND or no further treatment to the axilla [14]. The aim of this study was to analyze results of the first 1000 patients included in this trial, mainly for adjuvant treatments and non SN (NSN) involvement at cALND.

## Methods

### Study design and patients

SERC trial is a multicenter prospective randomized non-inferiority phase-3 trial comparing no ALND with ALND in patients with breast cancer and metastases in the SN, with a stratification planned between SN macro-metastases and ITC or micro-metastases. This study is registered with ClinicalTrials.gov, number NCT01717131. The primary objective is to demonstrate that the hazard ratio (SLNB vs ALND) for disease free survival is significantly lower than the non-inferiority margin set to 1.25. A total number of 3000 patients with 588 events have been calculated in order to answer with an 85% power and an error risk of 5% [14].

The first 1000 patients randomized were recruited from 44 institutions over an accrual period of 41 months from July 2012 to December 2015. Women eligible for registration could be any age > = 18 years, provided they had no previous or concomitant malignancy, pure ductal carcinoma in situ, previous systemic therapy before SLNB, distant metastases, palpable axillary nodes.

Compared to previous randomized trials, patients with one or more positive SN, multicentric tumours, <=T2 N0, ITC or micro-metastases or macro-metastases with or without capsular effraction were allowed to participate. Neoadjuvant chemotherapy (NAC) with SLNB before chemotherapy, mastectomy or conservative breast surgery was permitted.

Whole breast irradiation (WBI) was recommended after conservative surgery with boost on tumor basin and post mastectomy radiotherapy (PMRT) was proposed according to guidelines used in each center and start 4 to 8 weeks after surgery or after the end of adjuvant chemotherapy (AC). No specific axillary radiotherapy was delivered and 2 tangential fields were recommended for chest or WBI with a total dose of 50 Grays at ICRU point with 25 fractions of 2 Grays during 5 weeks. AC and endocrine-therapy (ET) were proposed according to guidelines used in each center.

The protocol was approved by the institutional review boards of all participating centers, and all participants provided written informed consent. Data were collected from participating centers through electronic CRFs, and centralized at the Institute-Paoli-Calmettes Data-Center-Unit.

### Randomization

The patients were randomly assigned, via a centralized interactive voice-response system (IVRS), to receive (1,1 ratio) either ALND or SLNB alone. Randomization was done with a permuted block randomization scheme stratified by participating center and SN status.

### Procedures

SN detection could be performed by combined isotopic and colorimetric methods or only isotopic method with peri-tumoral or retro-areolar injection. The SN could be examined intra-operatively and ALND done during the operation to remove the primary tumor or post operatively and later second surgery done if randomly assigned to undergo ALND. Axillary ultra-sonography was a systematic recommended pre-operative exam but was not recorded in the trial.

All SN were entirely sectioned at 50–200 μm intervals and all sections were examined with hematoxylin and eosin staining (HES) by pathologists at each participating center. Cytokeratin immunostaining was used only when HES was negative. SN could be examined by one step nucleic acid amplification method. A lysate with CK19 mRNA copy number/μl ranging between 250 and 5000 was classified as micro-metastases and greater than 5000 as macro-metastases.

### Statistical analysis

The cut-off date for data collection was May 29, 2016. Of the first 1000 randomized patients, only patients with monitoring review of eligibility data were included in the analysis set. Graphical display of cumulative numbers of accruals since study start, total accruals per participating centers were presented. All patients are grouped according to the treatment they had actually received (ALND or SLNB alone). Descriptive summaries of individual data (age, SBR grade, tumor histology, tumor size, lympho-vascular involvement (LVI), SN status, hormonal receptors (HR), tumor sub types) and treatment characteristics (radiotherapy and endocrine therapy (ET)) in the ALND and SLNB groups are presented as mean ± standard deviation, median (interquartile range) for continuous data, frequency (percent) for categorical data in the full analysis set and in the ALND and SLNB groups. Differences between actually-received treatment groups were evaluated using Mann–Whitney U and Chi-square tests as appropriate. Summary data according to the status of SN were also reported. AC rates were analyzed in the full analysis cohort and in both ALND and SLNB groups. NSN involvement rates were analyzed for patients with ALND in order to determine significant factors correlated with AC administration and NSN involvement. Univariate associations between patient, tumor, SLNB and treatment characteristics were assessed using logistic regression analysis. Multivariate logistic regression analysis was further used to identify independent factors associated with AC administration in HR positive patients and NSN involvement in patients treated by radiotherapy and without NAC.

All statistical analyses were carried out using SAS-Software (Release 9.3, SAS-Institute, Inc., Cary, NC). The level of statistical significance was set to 0.05, with no adjustment for multiplicity.

## Results

### Patient accruals and characteristics

**Fig. 1:**
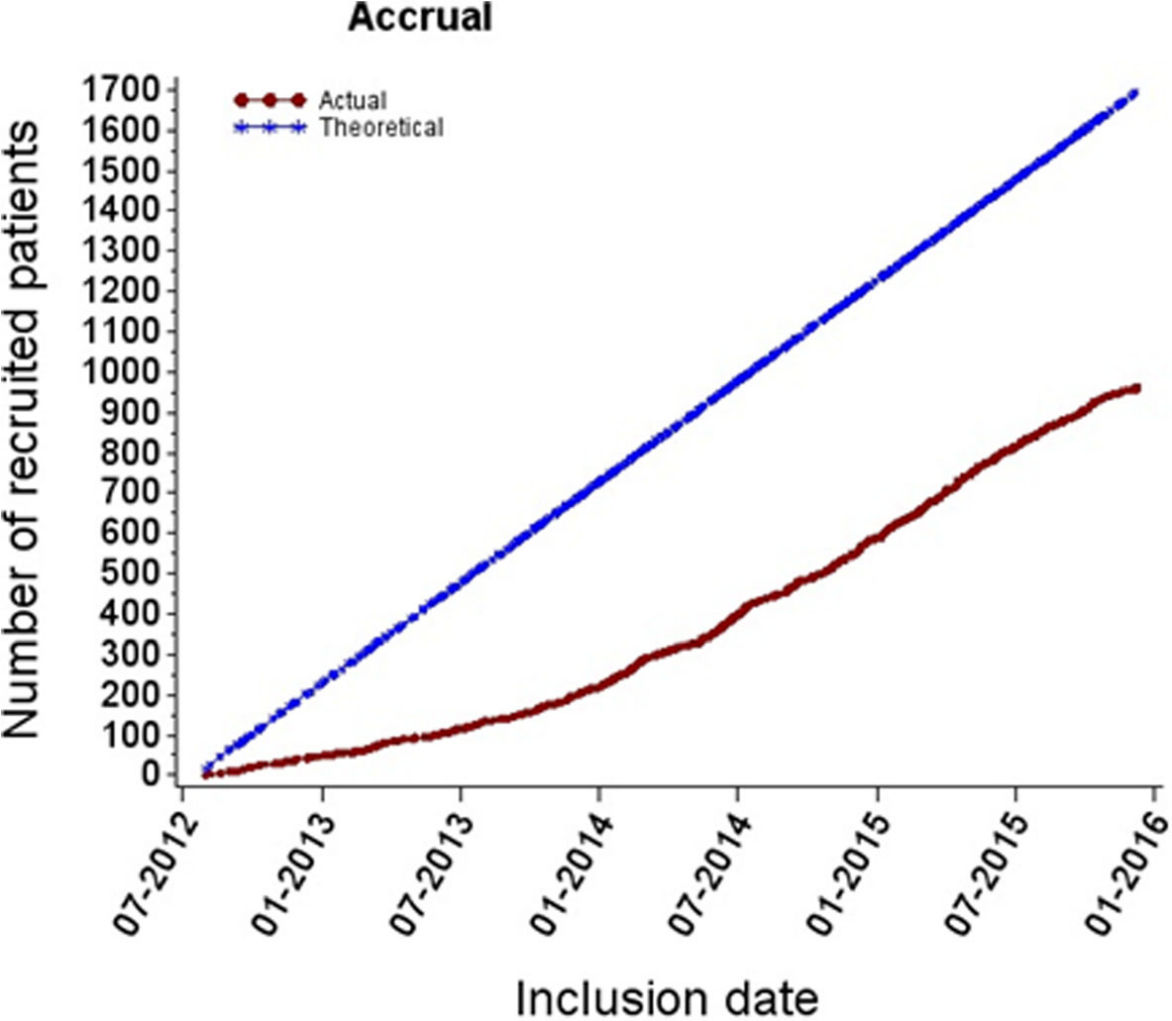
Observed vs. expected cumulative accrual numbers during (20/07/2012–09/12/2015)

**Fig. 2:**
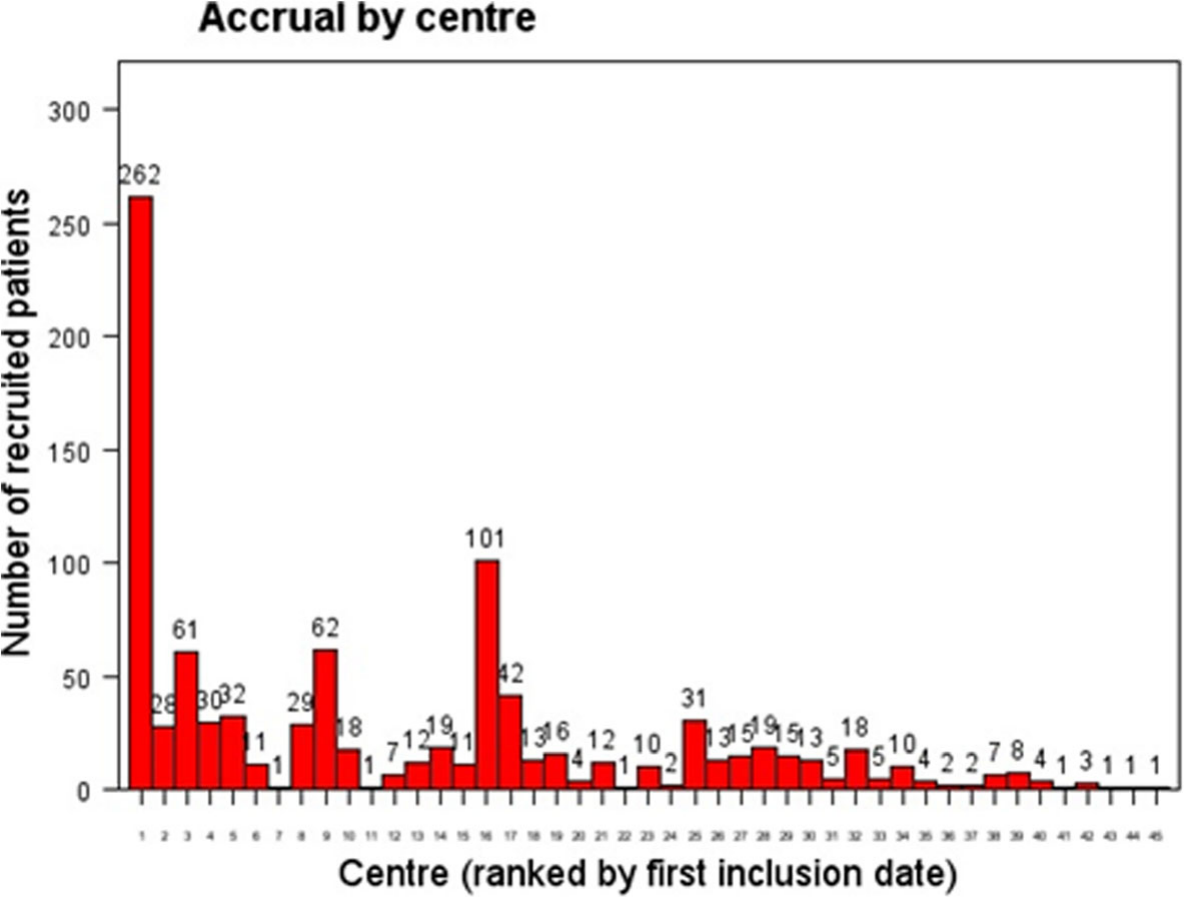
Number of patients accrued by participating center during the accrual period of first 1000 randomized patients: The majority of patients (528) were recruited from 4 different institutions and 20 centers accrued less than 10 patients

The first 1000 randomized patients were accrued from 44 centers (Fig. 1). The number of patients included in each center ranged between 1 to 262 (Fig. 2). Thirty-seven patients were excluded from the analysis set because of CRF empty (6) or incomplete monitoring (31). Of the 963 patients included in the analysis set, 478 were randomized to receive cALND and 485 SLNB alone. All patient demographics and tumor characteristics were balanced between the two arms (data not shown). Overall, median age was 58 years old (CI95% = 57.6–59), median tumor size was 18 mm (CI95% = 19–20.4), median number of harvested SN was 2 (689 < =2, 272 > 2) and median number of involved-SN was 1 (925 < =2, 14 > 2). The status of involved-SN was not determined in 60 patients (6.2%). SN ITC were present in 6.31%, micro-metastases in 33.0% and macro-metastases in 60.7%. Of the 846 patients with SN micro or macro-metastases, 289 (34.2%) were non-eligible to Z0011 criteria: capsular effraction (*n* = 157), mastectomy (*n* = 145), NAC (*n* = 25), > 2 involved SN (*n* = 13). Of the 355 patients with SN ITC or micro-metastases, 11 did not meet the eligibility criteria of IBCSG-23-01: NAC (*n* = 8), > 2 involved SN (n = 8) and tumor size (n = 1).

Forty-nine patients (4.9%) did not receive the study treatment as randomized: 42 (8.79%) in the ALND-arm did not have ALND and 7 (1.44%) in the SLNB-alone-arm had ALND with in summary, 443 who received cALND and 520 SLNB-alone. These protocol deviations were in relation with patient’s decision in 41 cases (7 for ITC SN, 14 micro-metastases, 18 macro-metastases and 2 unknown SN-status) and in relation with investigator’s decision in 2 cases (1 ITC, 1 pN1mi) to avoid cALND in ALND arm, with patient’s decision in 1 case (SN macro-metastases), with investigator’s decision in 5 cases (1 micro-metastases, 4 macro-metastases) and 2 cases without precision (1 SN macro-metastases and 1 unknown SN-status) to performed cALND in SLNB arm. No significant differences in patient’s characteristics (age, grade, tumor histology, tumor size, LVI, SN-status, HR and tumor sub types) and adjuvant treatment (AC, radiotherapy, ET, trastuzumab and type of surgery) were observed between the two actual treatment groups (Tables 1 and 2).

**Table 1:** Baseline patient, tumor and prior surgery treatment characteristics according to actually received study group (ALND vs SLNB)

Test	Class	All (*n* = 963)	ALND (*n* = 443)	SLNB alone (*n* = 520)	*p*-value
Age	Median [range]	58 [26–84]	59 [33–84]	58 [26–84]	0.761
	<= 40	51 (5.30)	26 (5.87)	25 (4.81)	0.7142
	41–75	858 (89.10)	391 (88.26)	467 (89.81)	
	> 75	54 (5.61)	26 (5.87)	28 (5.38)	
Clinical T stage	T1	571(59.60)	261(59.32)	310(59.85)	0.9393
	T2	342 (35.70)	156 (35.45)	186 (35.91)	
	T3	17 (1.77)	9 (2.05)	8 (1.54)	
	T4	5 (0.52)	2 (0.45)	3 (0.58)	
	Tis/Tx	23 (2.40)	12 (2.73)	11 (2.12)	
Tumor size (mm)	Median [range]	18 [0–81]	18 [0–80]	18 [0–81]	0.573
	<=10	151 (15.83)	64 (14.61)	87 (16.86)	0.5097
	10–30	704 (73.79)	331 (75.57)	373 (72.29)	
	> 30	99 (10.38)	43 (9.82)	56 (10.85)	
Histology Type	Infiltrating ductal	781(81.10)	358(80.81)	423(81.35)	0.8805
	Infiltrating lobular	102 (10.59)	45 (10.16)	57 (10.96)	
	Mixed	26 (2.70)	13 (2.93)	13 (2.50)	
	Other	54 (5.61)	27 (6.09)	27 (5.19)	
SBR Grade	Gr I	213 (22.76)	101 (23.60)	112 (22.05)	0.2677
	Gr II	479 (51.18)	207 (48.36)	272 (53.54)	
	Gr III	244 (26.07)	120 (28.04)	124 (24.41)	
LVI	No	281 (30.38)	134 (31.60)	147 (29.34)	0.4560
	Yes	644 (69.62)	290 (68.40)	354 (70.66)	
Capsular Effraction	No	698 (80.14)	327 (80.74)	371 (79.61)	0.6775
	Yes	173 (19.86)	78 (19.26)	95 (20.39)	
Hormonal receptors status	Negative	84 (8.89)	44 (10.16)	40 (7.81)	0.2061
	Positive	861 (91.11)	389 (89.84)	472 (92.19)	
HER2 status	Negative	815 (87.73)	369 (86.42)	446 (88.84)	0.26106
	Positive	114 (12.27)	58 (13.58)	56 (11.16)	
Tumor subtype (RH/HER2)	RH+/HER2+	83 (9.00)	41 (9.72)	42 (8.40)	0.5150
	RH+/HER2-	759 (82.32)	339 (80.33)	420 (84.00)	
	RH-/HER2+	30 (3.25)	16 (3.79)	14 (2.80)	
	RH-/HER2-	50 (5.42)	26 (6.16)	24 (4.80)	
Harvested SN, number	Median [range]	2[0–8]	2[0–7]	2[1–8]	0.996
	0	2 (0.21)	2 (0.45)		0.2823
	1	373 (38.73)	168 (37.92)	205 (39.42)	
	> 2	588 (61.06)	273 (61.63)	315 (60.58)	
	Median [range]	1[0–4]	1[0–4]	1[0–3]	0.422
Involved SN	<=2	925 (98.51)	424 (98.60)	501 (98.43)	0.8242
	> 2	14 (1.49)	6 (1.40)	8 (1.57)	
	ITC	57 (6.31)	22 (5.30)	35 (7.17)	0.5040
SN status	Micro	298 (33.00)	137 (33.01)	161 (32.99)	
	Macro	548 (60.69)	256 (61.69)	292 (59.84)	

*P* value based on Wilcoxon rank sum test for continuous variables or Chi square test for categorical variables

**Table 2:** Surgical and adjuvant treatment characteristics according to actually received study group (ALND vs SLNB)

Test	Classes	All (*n* = 963)	ALND (*n* = 443)	SLNB (*n* = 520)	*p*-value
Breast surgery Type	Mastectomy	170 (17.65)	77 (17.38)	93 (17.88)	0.8383
	Conservative	793 (82.35)	366 (82.62)	427 (82.12)	
Radiotherapy	No	38 (4.07)	19 (4.45)	19 (3.75)	0.5885
	Yes	896 (95.93)	408 (95.55)	488 (96.25)	
Chemotherapy	No	282 (29.59)	126 (28.70)	156 (30.35)	0.3482
	Neoadjuvant	27 (2.83)	16 (3.64)	11 (2.14)	
	Adjuvant	644 (67.58)	297 (67.65)	347 (67.51)	
Endocrine therapy	No	78 (10.39)	30 (8.72)	48 (11.79)	0.1691
	Yes	673 (89.61)	314 (91.28)	359 (88.21)	
Trastuzumab	No	578 (85.76)	261 (85.02)	317 (86.38)	0.6149
	Yes	96 (14.24)	46 (14.98)	50 (13.62)	

*P* value based on Wilcoxon rank sum test for continuous variables or Chi square test for categorical variables

### Adjuvant treatments

Full treatment information was not available in patients who did not complete the full sequence of treatment at the date of last follow-up for this analysis. Whole breast irradiation or PMRT was delivered in 95.9% of patients, including 134 PMRT (82.7%: 134/162): 94.8% (92/97) for macro-metastatic SN and 61.8% (34/55) for ITC or micro-metastases. PMRT rate was not significantly different between two arms: 82.7% (72/87) without ALND and 82.7% (62/75) with ALND. AC was administered in 69.5% of patients (644/926), ET in 89.6% and the proportions were similar in the two actual treatment groups: 237 of 423 (70.2%) in the ALND group and 347 of 503 (69.0%) in the SLNB alone received AC, 314 of 344 (91.3%) in the ALND group and 359 of 407 (88.2%) in the SLNB alone received ET. Patients, tumor and treatment characteristics (age, grade, tumor histology, tumor size, LVI, SN status, HR, tumor sub types, ET and radiotherapy) were significantly different in univariate analysis according to chemotherapy administration or not, without evidence of any difference in crudes rates of AC between ALND and SLNB groups (Table 3).

**Table 3:** Baseline patient, tumor and treatment characteristics according to adjuvant chemotherapy administration

Test		All			ALND		SLNB		SLBN vs ALND	
	Category	*N*	*n* (%)	*p* value^*^	*N*	*n* (%)	*N*	*n* (%)	Odd Ratio95%CI	*p* value^**^
Age in class	<= 40	45	44(.98)	<.001	22	21(.95)	23	23(1.0)	> 999[< 0.01,> 999]	0.978
	41–75	828	582(.70)		376	266(.71)	452	316(.70)	0.96[0.71, 1.30]	0.794
	> 75	53	18(.34)		25	10(.40)	28	8(.29)	0.61[0.19,1.88]	0.387
Tumor size (mm)	<=10	143	64(.45)		57	27(.47)	86	37(.43)	0.84[0.43,1.64]	0.610
	10–30	684	506(.74)		323	236(.73)	361	270(.75)	1.09[0.78,1.54]	0.608
	> 30	96	73(.76)	<.001	41	34(.83)	55	39(.71)	0.51[0.19,1.37]	0.179
Histology type	Infiltrant ductal	748	543(.73)		340	246(.72)	408	297(.73)	1.02[0.74,1.41]	0.893
	Infiltrant lobular	101	50(.50)		45	25(.56)	56	25(.45)	0.65[0.30,1.42]	0.279
	Mixed	25	19(.76)	<.001	12	10(.83)	13	9(.69)	0.46[0.07,3.05]	0.425
	Other	52	32(.62)		26	16(.62)	26	16(.62)	1.00[0.33,3.02]	1.000
SBR grade	Gr I	206	88(.43)	<.001	98	40(.41)	108	48(.44)	1.16[0.67,2.01]	0.600
	Gr II	464	317(.68)		200	139(.70)	264	178(.67)	0.91[0.61,1.35]	0.635
	Gr III	237	223(.94)		116	110(.95)	121	113(.93)	0.77[0.26,2.29]	0.640
LVI	No	275	235(.85)	<.001	130	110(.85)	145	125(.86)	1.14[0.58,2.22]	0.709
	Yes	616	394(.64)		276	178(.64)	340	216(.64)	0.96[0.69,1.34]	0.805
Capsular Effraction	No	673	467(.69)		313	217(.69)	360	250(.69)	1.01[0.72,1.40]	0.974
	Yes	167	122(.73)	0.355	75	56(.75)	92	66(.72)	0.86[0.43,1.72]	0.673
Hormonal receptors status	Negative	75	73(.97)	<.001	39	38(.97)	36	35(.97)	0.92[0.06,15.02]	0.955
	Positive	837	568(.68)		377	257(.68)	460	311(.68)	0.97[0.73,1.30]	0.863
HER2 status	Negative	794	532(.67)		358	242(.68)	436	290(.67)	0.95[0.71,1.28]	0.747
	Positive	102	98(.96)	<.001	52	50(.96)	50	48(.96)	0.96[0.13,7.02]	0.968
Tumor subtype (RH/HER2)	RH+/HER2+	75	72(.96)		36	35(.97)	39	37(.95)	0.53[0.05,6.05]	0.612
	RH+/HER2-	743	486(.65)		332	218(.66)	411	268(.65)	0.98[0.72,1.33]	0.897
	RH-/HER2+	26	26(1.0)	<.001	15	15(1.0)	11	11(1.0)		
	RH-/HER2-	45	44(.98)		22	22(1.0)	23	22(.96)	< 0.01[< 0.01,> 999.99]	0.978
Harvested SN, number	=1	365	253(.69)		165	115(.70)	200	138(.69)	0.97[0.62,1.51]	0.886
	=2	303	205(.68)		142	96(.68)	161	109(.68)	1.00[0.62,1.63]	0.986
	> 2	258	186(.72)	0.519	116	86(.74)	142	100(.70)	0.83[0.48,1.44]	0.509
Involved SN	<=2	891	619(.69)		406	285(.70)	485	334(.69)	0.94[0.71,1.25]	0.668
	> 2	14	13(.93)	0.059	6	6(1.0)	8	7(.88)	< 0.01[< 0.01,> 999.99]	0.979
SN status	ITC	56	31(.55)	<.001	22	14(.64)	34	17(.50)	0.58[0.19,1.71]	0.322
	Micro	289	164(.57)		133	76(.57)	156	88(.56)	0.97[0.61,1.55]	0.900
	Macro	525	405(.77)		241	183(.76)	284	222(.78)	1.13[0.75,1.71]	0.544
Endocrine therapy	No	75	66(.88)	<.001	29	28(.97)	46	38(.83)	0.17[0.02,1.45]	0.106
	Yes	653	420(.64)		302	192(.64)	351	228(.65)	1.06[0.77,1.46]	0.714
Trastuzumab	No	567	368(.65)		256	162(.63)	311	206(.66)	1.14[0.81,1.61]	0.463
	Yes	83	83(1.0)	<.001	38	38(1.0)	45	45(1.0)		
Breast Surgery	Mastectomy	160	120(.75)	0.099	73	55(.75)	87	65(.75)	0.97[0.47,1.98]	0.927
	Conservative	766	524(.68)		350	242(.69)	416	282(.68)	0.94[0.69,1.28]	0.688
Radiotherapy	No	38	19(.50)	0.010	19	10(.53)	19	9(.47)	0.81[0.23,2.86]	0.749
	Yes	867	604(.70)		390	273(.70)	477	331(.69)	0.97[0.73,1.30]	0.846

*P value derived from Chi square test for categorical variables

**P value derived from analysis including treatment group as received as single factor in logistic regression

Abbreviations: *ALND* axillairy lymph node dissection, *SLNB* sentinel lymph node dissection alone, *CI* confidence interval

AC was administered in 97% for patients with HR- tumors (73/75), 67.7% for patients with HR+ tumors (568/837), 55.4% for patients with SN involved by ITC (31/56), 56.8% for SN micro-metastases (164/289), 77.1% for SN macro-metastases (405/525). Among HR+ patients, multivariate analysis identified age, tumor size, LVI, HER2 status, grade and SN status as significant predictors of AC. Chemotherapy use increased for: tumors with size 11-30 mm (OR = 2.72) or > 30 mm (OR: 2.65), ductal carcinoma (OR = 2.10), tumors with LVI (OR = 2.16), SBR grade 2 (OR = 3.05) or grade 3 (OR = 25.95), HER2+ (OR = 3.12), SN macro-metastases (OR = 3.70), and for women age between 41 and 75 years old (OR = 13.9) or < =40 years (OR = 75.8) (Table 4). Forty patients had involved SN > 2: all presented HR+ tumors and one did not received AC.

**Table 4:** Multivariate analysis of factors associated with chemotherapy administration in hormone-receptor positive patients

Effect	*P* value	Contrast	Odd Ratio Estimate	95% CI	*P* value
Age in class	<.0001	41–75 vs > 75	13.894	[5.050; 38.228]	<.0001
		<=40 vs > 75	75.801	[14.803; 388.145]	<.0001
Tumor Size	0.0017	10–30 vs < =10	2.724	[1.567; 4.737]	0.0004
		> 30 vs < =10	2.654	[1.060; 6.646]	0.0372
LVI	0.0036	Pos. Vs Neg.	2.163	[1.287; 3.634]	0.0036
HER2 Status	0.0470	Pos. Vs Neg.	3.120	[1.015; 9.588]	0.0470
Histology Type	0.0930	Mixed/Other vs lobular	1.866	[0.716; 4.863]	0.2020
		Ductal vs lobular	2.096	[1.077; 4.077]	0.0293
SBR Grade	<.0001	Gr II vs Gr I	3.052	[1.875; 4.967]	<.0001
		Gr III vs Gr I	25.946	[10.448; 64.435]	<.0001
Nb Involved SN	0.4193	> 2 vs < =2	2.635	[0.251; 27.672]	0.4193
SN Status	<.0001	Macro vs ITC	3.695	[1.571; 8.692]	0.0027
		Micro vs ITC	1.447	[0.603; 3.474]	0.4078
Surgery	0.2921	Conservative vs Mastectomy	0.715	[0.382; 1.335]	0.2921
Hormonotherapy	0.6144	Yes vs No	1.429	[0.357; 5.721]	0.6144
Radiotherapy	0.3787	Yes vs No	1.636	[0.547; 4.899]	0.3787
ALND status	0.5602	Yes vs No	0.881	[0.574; 1.350]	0.5602

Abbreviations: *ALND* axilliary lymph node dissection, *SLNB* sentinel lymph node dissection alone, *CI* confidence interval

### Final pathological findings in ALND group

Of the 443 patients who underwent ALND, the number of involved NSN was reported missing in one patient. The overall rate of positive NSN was 19% for patients with cALND. Crude rates of positive NSN according to SN status were 4.5% for patients with ITC (1/22), 9.5% for SN micro-metastases (13/137), 23.9% for SN macro-metastases (61/255). Univariate analysis of patient and tumor characteristics revealed that positive NSN rates were significantly higher for patients with tumor sizes > 10 mm, > 2 involved SN, macro-metastatic SN, presence of LVI and SN capsular effraction (Table 5). Crude rates of positive NSN rates were significantly higher in patients who received radiotherapy or chemotherapy. Of the 19 patients in the ALND group who did not received radiotherapy, none reported positive NSN. Crude rates of positive NSN were 7.9% for patients without systemic therapy (13/142), 18.8% with NAC (3/16) and 23.9% with AC (71/297).

**Table 5:** Baseline patient, tumor and prior surgery treatment characteristics according to non sentinel Node (NSN) involvement

Test			Non sentinel node involvment		
	Class	All (*n* = 443)	No (*n* = 358)	Yes (*n* = 84)	*p-*value
Age	Median [range]	59 [33–84]	59 [33–84]	59 [39–84]	0.828
	<= 40	26 (5.87)	24 (6.70)	2 (2.38)	0.2864
	41–75	391 (88.26)	314 (87.71)	76 (90.48)	
	> 75	26 (5.87)	20 (5.59)	6 (7.14)	
Clinical T stage	T1	261 (59.32)	216 (60.67)	44 (53.01)	0.2384
	T2	156 (35.45)	123 (34.55)	33 (39.76)	
	T3	9 (2.05)	5 (1.40)	4 (4.82)	
	T4	2 (0.45)	2 (0.56)		
	Tis/Tx	12 (2.73)	10 (2.81)	2 (2.41)	
Tumor size (mm)	Median [range]	18 [0–80]	18 [0–70]	19 [0–80]	0.059
	<=10	64 (14.61)	55 (15.58)	9 (10.71)	0.0465
	10–30	331 (75.57)	269 (76.20)	61 (72.62)	
	> 30	43 (9.82)	29 (8.22)	14 (16.67)	
Histology Type	Infiltrating ductal	358 (80.81)	290 (81.01)	67 (79.76)	0.4021
	Infiltrating lobular	45 (10.16)	33 (9.22)	12 (14.29)	
	Mixed	13 (2.93)	11 (3.07)	2 (2.38)	
	Other	27 (6.09)	24 (6.70)	3 (3.57)	
SBR Grade	Gr I	101 (23.60)	88 (25.51)	12 (14.63)	0.1104
	Gr II	207 (48.36)	162 (46.96)	45 (54.88)	
	Gr III	120 (28.04)	95 (27.54)	25 (30.49)	
LVI	No	134 (31.60)	100 (29.41)	34 (40.96)	0.0425
	Yes	290 (68.40)	240 (70.59)	49 (59.04)	
Capsular Effraction	No	327 (80.74)	276 (83.64)	50 (67.57)	0.0016
	Yes	78 (19.26)	54 (16.36)	24 (32.43)	
Hormonal receptors	Negative	44 (10.16)	36 (10.32)	8 (9.64)	0.8546
	Positive	389 (89.84)	313 (89.68)	75 (90.36)	
HER2 status	Negative	369 (86.42)	297 (86.34)	71 (86.59)	0.9530
	Positive	58 (13.58)	47 (13.66)	11 (13.41)	
Tumor subtype (RH/HER2)	RH+/HER2+	41 (9.72)	34 (10.00)	7 (8.64)	0.8656
	RH+/HER2-	339 (80.33)	272 (80.00)	66 (81.48)	
	RH-/HER2+	16 (3.79)	12 (3.53)	4 (4.94)	
	RH-/HER2-	26 (6.16)	22 (6.47)	4 (4.94)	
Harvested SN, number	Median [range]	2 [0–7]	2 [0–7]	2 [0–5]	0.650
	0	2 (0.45)	1 (0.28)	1 (1.19)	0.4885
	1	168 (37.92)	138 (38.55)	30 (35.71)	
	> 2	273 (61.63)	219 (61.17)	53 (63.10)	
Involved SN	Median [range]	1 [0–4]	1 [0–4]	1 [0–4]	0.004
	<=2	424 (98.60)	346 (99.43)	77 (95.06)	0.0026
	> 2	6 (1.40)	2 (0.57)	4 (4.94)	
	ITC	22 (5.30)	21 (6.19)	1 (1.33)	0.0005
SN status	Micro	137 (33.01)	124 (36.58)	13 (17.33)	
	Macro	256 (61.69)	194 (57.23)	61 (81.33)	
	Mastectomie	77 (17.38)	60 (16.76)	17 (20.24)	0.4494
Breast surgery Type	Conservative	366 (82.62)	298 (83.24)	67 (79.76)	
	No	19 (4.45)	19 (5.51)		0.0297
Radiotherapy	Yes	408 (95.55)	326 (94.49)	82 (100.0)	
	No	126 (28.70)	116 (32.68)	10 (11.90)	0.0007
Chemotherapy	Neoadjuvant	16 (3.64)	13 (3.66)	3 (3.57)	
	Adjuvant	297 (67.65)	226 (63.66)	71 (84.52)	
	No	30 (8.72)	25 (9.06)	5 (7.35)	0.6553
Endocrine therapy	Yes	314 (91.28)	251 (90.94)	63 (92.65)	
	No	261 (85.02)	208 (84.90)	53 (85.48)	0.9081
Trastuzumab	Yes	46 (14.98)	37 (15.10)	9 (14.52)	

Systemic therapy was administered in 88.1% for patients with involved NSN (74/84) of whom 11.9% with chemotherapy first administrated prior to ALND (10/84) and 76.2% with chemotherapy administered after ALND (64/84). As ALND omission precludes the observation of positive NSN, logistic regression was used to identify non determinant factors of AC predictive of NSN involvement. In addition to patients who received NAC, patients who did not receive radiotherapy were excluded due the lack of positive NSN. Multivariate regression analysis taking into account key determinant factors of AC identified capsular effraction (OR = 2.31, *p* = 0.028) and involved SN > 2(OR = 8.16, *p* = 0.081) as significant or of borderline significance predictors of NSN involvement (Table 6**)**. Multivariate analysis excluding patients who received chemotherapy prior to ALND lead to the same conclusions (capsular effraction: OR = 2.36, *p* = 0.038; involved SN > 2: OR = 6.78, *p* = 0.12) (Table 7).

**Table 6:** Multivariate analysis of factors associated with NSN involvement in patients treated by radiotherapy and without neoadjuvant chemotherapy

Effect	*P* value	Contrast	Odd Ratio Estimate	95% CI	*P* value
Age in class	0.2643	41–75 vs > 75	1.491	[0.448; 4.963]	0.5148
		<=40 vs > 75	0.294	[0.027; 3.174]	0.3132
Tumor Size	0.9532	10–30 vs < =10	0.852	[0.309; 2.352]	0.7574
		> 30 vs < =10	0.869	[0.218; 3.469]	0.8420
LVI	0.4760	Pos. Vs Neg.	0.781	[0.396; 1.540]	0.4760
Capsular Effraction	0.0227	Yes vs No	2.311	[1.124; 4.753]	0.0227
Hormonal receptors	0.7739	Pos. Vs Neg.	1.206	[0.336; 4.323]	0.7739
HER2 Status	0.1481	Pos. Vs Neg.	0.406	[0.119; 1.378]	0.1481
Histology Type	0.3399	Mixed/Other vs lobular	0.343	[0.082; 1.430]	0.1418
		Ductal vs lobular	0.644	[0.244; 1.699]	0.3739
SBR Grade	0.1191	Gr II vs Gr I	2.672	[1.048; 6.817]	0.0396
		Gr III vs Gr I	2.179	[0.738; 6.436]	0.1586
Nb Involved SN	0.0808	> 2 vs < =2	8.159	[0.773; 86.095]	0.0808
SN Status	0.3649	Macro vs ITC	3.303	[0.404; 27.011]	0.2651
		Micro vs ITC	2.247	[0.262; 19.277]	0.4603

Abbreviations: *CI* confidence interval

**Table 7:** Multivariate analysis of factors associated with NSN involvement in patients treated by radiotherapy and without chemotherapy prior to ALND

Effect	*P* value	Contrast	Odd Ratio Estimate	95% CI	*P* value
Age in class	0.2626	41–75 vs > 75	1.402	[0.409; 4.805]	0.5905
		<=40 vs > 75	0.256	[0.023; 2.887]	0.2702
Tumor Size	0.9233	10–30 vs < =10	0.842	[0.273; 2.598]	0.7644
		> 30 vs < =10	0.982	[0.226; 4.263]	0.9807
LVI	0.3089	Pos. Vs Neg.	0.682	[0.326; 1.425]	0.3089
Capsular Effraction	0.0381	Yes vs No	2.357	[1.048; 5.302]	0.0381
Hormonal receptors	0.4821	Pos. Vs Neg.	1.647	[0.410; 6.617]	0.4821
HER2 Status	0.1906	Pos. Vs Neg.	0.376	[0.087; 1.626]	0.1906
Histology Type	0.5892	Mixed/Other vs lobular	0.478	[0.111; 2.047]	0.3196
		Ductal vs lobular	0.665	[0.243; 1.818]	0.4267
SBR Grade	0.0650	Gr II vs Gr I	3.332	[1.214; 9.143]	0.0195
		Gr III vs Gr I	2.714	[0.831; 8.862]	0.0982
Nb Involved SN	0.1171	> 2 vs < =2	6.797	[0.618; 74.708]	0.1171
SN Status	0.3734	Macro vs ITC	3.737	[0.451; 30.991]	0.2219
		Micro vs ITC	2.635	[0.305; 22.767]	0.3784

Abbreviations: *CI* confidence interval

For patients with cALND (*n* = 419), involved NSN rates were respectively 29.36% (64/218) when chemotherapy was administered after cALND, 9.33% (7/75) when chemotherapy was administered before cALND and 7.94% (10/126) for patients without AC. For SN ITC, involved NSN rate was 4.5% (1/22) with chemotherapy administered after ALND. Involved NSN rates were respectively 5.3% (3/57) without chemotherapy, 0% (0/17) with chemotherapy administered before ALND, 21.7% (10/46) with chemotherapy administered after ALND for SN micro-metastases and respectively 12.0% (7/58), 15.5% (7/45) and 51.8% (44/85) for SN macro-metastases.

We reported only one positive NSN in 49 patients (58.3%), 2 positive NSN in 9 (10.7%) and 3 or more in 26 (31.0%), respectively 64.3% (9/14), 21.4% (3/14) and 14.3% (2/14) for involved SN by ITC or micro-metastases and 55.7% (34/61), 6.6% (4/61) and 37.7 (23/61) for SN macro-metastases. Number of positive NSN according to administration time of AC were respectively for no chemotherapy, ALND before chemotherapy and ALND after chemotherapy: only one positive NSN in 60% (6/10), 54.7% (35/64) and 71.4% (5/7) patients, 2 positive NSN in 20% (2/10), 9.4% (6/64) and 14.3% (1/7) patients and > 3 positive NSN in 20% (2/10), 35.9% (23/64) and 14.3% (1/7) patients.

For 256 patients non eligible to Z0011 with SN macro-metastases in all patients, 33 (30.3%: 33/109) had involved NSN, respectively 19, 3 and 11 with 1, 2 and > 3 positive NSN. When cALND was performed after chemotherapy, positive NSN rate was 19.4% (6/31) in comparison with 48.1% (25/52) for patients with cALND performed before chemotherapy and 8.0% (2/25) with cALND without chemotherapy (*p* < 0.001). There was no difference between ALND and no ALND, respectively 109 and 116 patients (48.4 and 51.6%). Chemotherapy was delivered in 92.4% of these patients, ET in 91.5%, trastuzumab in 13.8%, radiotherapy in 96.8% and surgery was a total mastectomy in 44.9% with PMRT in 93.9%.

## Discussion

In this study 963 patients were evaluable and actually 1834 patients were included in SERC trial. Number of included patients was respectively 856, 931 and 233 in ACOSOG-Z0011 [10], IBCSG-23-01 [9] and AATRM trials [11], respectively from 177 institutions between May 2001 and December 2014, 27 institutions between April 2001 and February 2010, 18 institutions between January 2001 and December 2008. However, in Z0011 and IBCSG-23-01 less than 50% of patients were included in comparison with initial effective calculated to be able to demonstrate equivalence between the two arms. In AATRM trial, the sample size was estimated to be 352 patients and a maximum difference of 15% in disease free survival for the experimental group was established as clinically significant.

In SERC trial, about 287 patients were included per year and actually with 80 institutions about 340 patients per year are included. In comparison, for Z0011, IBCSG-23-01 and AATRM, numbers of patients included per year were respectively 63, 103 and 33.

In our study, 49 patients (4.9%) did not received the study treatment as randomized: 42 in the ALND arm did not have ALND (8.79%), and 7 in the SLND-alone arm had ALND (1.44%). Similar results were reported in Z0011 trial: 43 patients (5.0%) did not receive the study treatment as randomized: 32 in the ALND group (7.6%) and 11 in the SLND-alone group (2.5%). In IBCSG-23-01 trial, 31 patients (3.3%) did not receive the study treatment as randomized: 17 in the ALND arm (4%) and 14 in the SLND-alone arm (3%).

We reported in this study 548 patients (60.7%) with SN macro-metastases and 298 with micro-metastases (33.0%). Among 477 patients eligible in our study to Z0011 trial, we observed 276 macro-metastases (57.86%) and 201 micro-metastases (42.14%) in comparison with Z11 trial results, respectively 430 and 301 patients (50.2% and 35.2) but with undetermined SN status for 125 patients (14.6%). It was reported a significant difference of macro and micro-metastases between the two arms of the Z11 trial (44.8% of micro-metastases in no ALND arm versus 37.5% in ALND arm) with also 33 patients without involved SN, respectively 29 in no ALND arm and 4 in ALND arm. This point represents a strong limitation to demonstrated equivalent results between two arms.

Among 355 patients reported in our study, 340 (95.77%) were eligible to IBCSG-23-01 trial: 285 with micro-metastases (83.82%) and 55 with ITC (16.18%). We can’t compare this SN status with IBCSG-23-01 trial because this repartition was not done.

In our study 84 patients (8.89%) have negative ER and PR (< 10%) and 861 have positive ER or PR with 82.32% HR+ Her2- tumors, 5.42% triple negative tumors and 12.25% tumors Her2+ and HR- or HR+. Tumors subtypes were not done for randomized trials reported in literature. In Z11 trial 127 patients had ER and PR negative tumors (127/775: 16.4%), 91 had ER negative tumors (91/925: 9.8%) in IBCSG-23-01 trial and 28 had ER negative tumors (28/208: 13.5%) in AATRM trial.

In our study, involved NSN rate was 19%, respectively 15.7 and 12.8% for patients eligible to Z11 and IBCSG-23-01 trials, in comparison with 27.3, 7.6 and 13% for Z11, IBCSG-23-01 and AATRM trials. The difference (11.6%) observed between Z11 trial and our results for patients eligible to Z11 could be explain by the proportion of patients in our study with ALND performed after chemotherapy (17.9%: 75/419). Involved NSN rate is significantly lower for patients with ALND performed after chemotherapy, with a significant down staging in comparison with others patients with chemotherapy after ALND or without chemotherapy. Similar results were observed after NAC with a 41% related NAC down staging in ACOSOG-Z1071 trial [15], 17.8% positive NSN rate for patients cN0 with positive SN before NAC (SENTINA trial) [16] and 40.8% related NAC down staging for patients with positive cytology axillary node before NAC in Park et al. study [17]. This is an important observation which can in part explain the very low axillary recurrence rate for patients without cALND. A high proportion of patients received chemotherapy: 70.4% (671/953) in our study, 57.9% (496/856) in Z0011, 69.4% (646/931) in IBCSG-23-01 and 92.1% (199/216) in AATRM.

Tangential fields of breast irradiation have also a therapeutic impact on axillary basin as we can observe with an 10-year axillary recurrence rate of 0.08 and 0.75% respectively for WBI and partial breast irradiation (HR 0,25: 0,08-0,75) [18]. In our study 95.9% (896/934) patients received radiotherapy on the breast or chest wall, with respectively 89.3% (540/605) in Z11, 89.7% (209/215 after conservative treatment) in AATRM and 80.6% (661/820 WBI after breast conserving surgery) in IBCSG-23-01. Modality of radiotherapy is a key point in these randomized studies. In Z11, detailed radiotherapy records were obtained for only 228 patients (26.6%), of whom 185 (81.1%) received tangent-only treatment and 43 (18.9%) received directed regional nodal radiotherapy using 3 or more fields for patients with greater nodal involvement (55.6% for 3 nodes involved and 81.3% for > 4 nodes involved) and more often for patients without ALND (65%: 13/20 in the ALND arm versus 100%: 5/5 in the SLND arm for patients with > 3 involved nodes) [19]. This point, with about 20% of patients who received directed regional axillary radiotherapy, represents a strong limitation to demonstrated equivalent results between two arms.

Moreover, ET can also have a therapeutic impact on axillary lymph nodes [20] and was often performed: 46.5% (398/856) for Z11, 87.8% (817/931) for IBCSG-23-01, 61.6% (133/216) in AATRM and 89.61% (673/751) in our study.

Total mastectomy was done for 86 patients (9.2%) in IBCSG-23-01 trial, 18 (7.7%) in AATRM and 170 (17.65%) in our study. We reported total mastectomy for 16.6% (59/355) patients with SN involved by ITC or micro-metastases and 18.2% (100/548) for SN macro-metastases with chest wall irradiation respectively for 61.8% (34/55) and 93.9% (92/98) of patients. No data was reported about mastectomy with SN macro-metastases in previous randomized studies. We hope that results of SERC trial and BOOG 2013–07 trial [21] should be a sufficient rational to propose omission of cALND for patients with SN macro-metastases with mastectomy in the next future. Unfortunately, BOOG trial had to be shut down due to insufficient inclusion.

For 289 patient’s non eligible to Z11, we observed higher involved NSN rate in comparison with others patients and higher rate when cALND was performed before chemotherapy. This group of patients, which represent 30% of all patients in our study, was not included in previous randomized trials and received more adjuvant treatments.

Several non-inferiority trials with ALND randomization for involved SN by macro-metastases are ongoing [21–24] in order to confirm the possibility to avoid ALND, considering that previous trial’s results have a low level of evidence [25]. In AMAROS trial, there was no significant difference between two arms with ALND or axillary radiotherapy for patients with SN involvement, but authors discussed these results because no equivalent results could be achieved considering a smaller number of patients in this trial [26]. In SERC trial, we proposed to analyzed SN macro-metastases but also micro-metastases or ITC with a planned stratification between these SN metastases sizes, both to conservative treatment and mastectomy, because results for SN micro-metastases were not sufficient to consider that ALND omission is demonstrated with a high level of evidence, particularly in cases of mastectomy [27].

## Conclusion

The main objective of SERC trial is to demonstrate non inferiority of cALND omission to confirm or not previous randomized trials results for the same patients but also for others patients particularly for patients with capsular effraction or after mastectomy. A strong interaction between timing of cALND and chemotherapy with positive NSN rate was observed for different sizes of SN involvement.

Despite the declining trend to indicate cALND, the rate of inclusion since 2013 seems satisfactory, with however a heterogeneous participation of the centers, and could be improved by a better participation on a large number of centers. Actually, 1834 patients had been included in SERC trial.

## Additional file

Additional file 1: **Figure S1.** Wrong treatment received: A) according to time to study initiation. B) according to cumulative number of included patients. (PNG 32 kb)

## Abbreviations

AC: Adjuvant chemotherapy
ALND: Axillary lymph node dissection
cALND: Complementary axillary lymph node dissection
CRFs: Case report form
ET: Endocrine-therapy
HES: Hematoxylin and eosin staining
HR: Hormonal receptor
ITC: Isolated tumor cells
IVRS: Interactive voice-response system
LVI: Lympho vascular involvement
NAC: Neoadjuvant chemotherapy
NSN: Non-sentinel node
PMRT: Post mastectomy radiotherapy
SBR: Scarff-Bloom et Richardson
SERC: Sentinel Envahi Randomisaton Curage (essai de phase III multicentrique randomise de non-infériorité de la réalisation ou non du curage axillaire en cas d’envahissement du (des) ganglion(s) sentinelle(s) dans le cancer du sein invasif - SERC-IPC 2012–001)
SLNB: Sentinel lymph node biopsy
SN: Sentinel node
WBI: Whole breast irradiation
