# Supplementary figures and images for: Overview of the pathological results and treatment characteristics in the first 1000 patients randomized in the SERC trial: axillary dissection versus no axillary dissection in patients with involved sentinel node
Source: BMC Cancer. 2018 Nov 21;18:1153. doi: 10.1186/s12885-018-5053-7 (PMC6249981; doi:10.1186/s12885-018-5053-7)

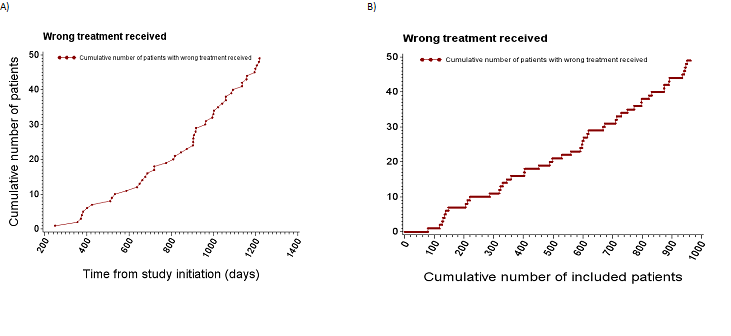

Supplement: Supplementary file 1 — Figure S1. Wrong treatment received: A) according to time to study initiation. B) according to cumulative number of included patients. (PNG 32 kb) [file 12885_2018_5053_MOESM1_ESM.png]
